# Targeting immunotherapy for bladder cancer using anti‐CD3× B7‐H3 bispecific antibody

**DOI:** 10.1002/cam4.1775

**Published:** 2018-09-25

**Authors:** Wanru Ma, Juan Ma, Ping Ma, Ting Lei, Man Zhao, Man Zhang

**Affiliations:** ^1^ Collage of Medical Technique Xuzhou Medical University Jiangsu China; ^2^ Department of Clinical Laboratory Medicine Beijing Shijitan Hospital Capital Medical University Beijing China; ^3^ Peking University Ninth School of Clinical Medical Beijing China; ^4^ Beijing Key Laboratory of Urinary Cellular Molecular Diagnostics Beijing China

**Keywords:** B7‐H3, bispecific antibody, bladder cancer, immunotherapy

## Abstract

**Objective:**

B7‐H3 is attractive for cancer immunotherapy with B7‐H3 overexpressed tumors. To explore whether B7‐H3 is an effective target for patients with bladder cancer, anti‐CD3× anti‐B7‐H3 bispecific antibodies (B7‐H3Bi‐Ab) was armed with activated T cells (ATC) to kill bladder cancer cells.

**Methods:**

High expressions of B7‐H3 on human bladder cancer cells were detected, including Pumc‐91 and T24 cells, and their chemotherapeutic drug‐resistant counterparts. ATC generated from healthy donors were stimulated with anti‐CD3 monoclonal antibody and interleukin‐2 (IL‐2) for 13 days. The ability of ATC armed with B7‐H3Bi‐Ab to kill bladder cancer cells was detected by flow cytometry, LDH, Elisa, and luciferase quantitative assay. Moreover, ATC generated from bladder cancer patients was armed with B7‐H3Bi‐Ab to verity the cell killing by the methods as previously described.

**Results:**

Compared with unarmed ATC, a significant increased cytotoxicity of B7‐H3Bi‐Ab‐armed ATC against bladder cancer cells was discovered. The B7‐H3Bi‐Ab‐armed ATC secreted more IFN‐γ, TNF‐α, and expressed high levels of activation marker CD69. Interestingly, despite the presence of immunosuppression in patients and resistance in chemotherapeutic drug‐resistant cancer cell lines, B7‐H3Bi‐Ab‐armed ATC from patients with bladder cancer still showed significant cytotoxic activity against bladder cancer cells and their chemotherapeutic drug‐resistant counterparts.

**Conclusion:**

B7‐H3 is an effective target for bladder cancer. B7‐H3Bi‐Ab enhances the ability of ATC to kill bladder cancer cells. B7‐H3Bi‐Ab‐armed ATC is promisingly to provide a novel strategy for current bladder cancer therapy.

## 1 INTRODUCTION

Bladder cancer is one of most common urinary tract cancers among people. In 2017, there are an estimated 79 030 cases of newly diagnosed bladder cancer and 19 870 deaths in the United States, with male morbidity and mortality four times higher than female.^1^ Superficial bladder cancer cases accounts for about 85% of bladder cancer and more than 45% of these patients have tumor recurrence and progression.^2^, ^3^, ^4^ Moreover, only 46% of the stage III patients and 15% of the stage IV patients can achieve a five‐year survival rate.^5^, ^6^ Despite great quantity treatment methods are used, for instance surgery, radiotherapy, and chemotherapy, the postoperative survival rate of bladder cancer is still very low.^7^

Immunotherapy is recognized as the fourth treatment in tumor comprehensive therapy strategy in the twenty‐first century.^8^ There are two ways to enhance anti‐tumor immunity. One is reducing immunosuppression by immunomodulation, vaccines, and targeting major immune checkpoint pathways, such as cytotoxic T‐lymphocyte‐associated antigen 4 (CTLA4), programmed cell death protein 1 (PD‐1)/PD‐1 ligand (PD‐L1), and Killer‐cell immunoglobulin‐like receptors (KIRs).^9^, ^10^ Applying bispecific antibodies (Bi‐Abs) to activated T cells (ATC) is also an effective strategy to improve antitumor activity.

With more than 15 mAbs clinically approved,the current overall immunotherapy effect is encouraging.^11^ B7‐H3, also known as CD276, has up to 30% same amino acid with the B7 family members.^11^ It is highly expressed in many kinds of cancer and has been shown to promote tumor development, including acute leukemia,^12^ glioma,^13^ hepatocellular, carcinoma,^14^ breast cancer,^15^ prostate cancer,^16^ osteosarcoma,^17^ skin melanoma,^18^ and pancreatic cancer.^19^ Liu et al^20^ discovered that the silence of B7‐H3 by lentivirus caused the increased sensitivity to gemcitabine in human pancreatic cancer cell line Patu8988 due to increased drug‐induced apoptosis. Ma et al^21^ synthesized anti‐CD3 x anti‐B7‐H3 bispecific antibody (B7‐H3Bi‐Ab) against B7‐H3+ tumor cell and observed an increased cytotoxic activity in B7‐H3Bi‐Ab‐armed ATC against some tumor cells. Moreover, through the PI3K/Akt/STAT3 signaling pathway, high expression of B7‐H3 promotes bladder cancer cells invade and metastasize.^22^ These results indicate that B7‐H3 probable be an efficacious target in the therapy of bladder cancer.

Here we proved the high expression of B7‐H3 on human bladder cancer cells. Anti‐CD3 antibody was conjugated with anti‐B7‐H3 antibody chemically, and ATC from both healthy donors and bladder cancer patients were armed with B7‐H3Bi‐Ab. Next the ability of B7‐H3Bi‐Ab‐armed ATC to kill bladder cancer cell and their chemotherapeutic drug‐resistant counterparts was explored. The B7‐H3Bi‐Ab‐armed ATC, with the higher expression of activation marker CD69, showed increased cytotoxicity and secreted more IFN‐γ and TNF‐α than unarmed ATC.

## 2 MATERIALS AND METHODS

### 2.1 Cell culture

The human bladder cancer pumc‐91 cell line was obtained from the Cell Laboratory of Beijing Union Medical College Hospital. The pumc‐91/ADM was a drug resistant cell line that was established by adding the dosage of Adriamycin. The final concentration of Adriamycin was 1.0 μg/mL.^23^, ^24^, ^25^, ^26^ The human bladder cancer T24 cell line was obtained from the Chinese Academy of Sciences. The drug resistant cell line was T24/DDP, which was established by increasing the dosage of cisplatin, and the final concentration of cisplatin was 0.6 μg/mL.^26^, ^27^ All the cell lines were cultured in RPMI 1640 medium with 15% fetal bovine serum and incubated in an incubator containing 5% carbon dioxide at 37°C.

### 2.2 Preparation and cryopreservation of activated T cells from peripheral blood lymphocytes

Peripheral blood mononuclear cells (PBMCs) were separated immediately by Ficoll‐Hypaque density gradient centrifugation. Blood was obtained from healthy people which provided by the Beijing Blood Bank. PBMCs were cultured at 1 × 10^6^/mL in RPMI‐1640 medium with 10% FBS. The ATC cells were stimulated by 5 μg/mL anti‐CD3 mAb (eBioscience, San Diego, CA, USA) and interleukin‐2 every. On day 13, the amplified ATC of healthy donors were 98.34% CD3+ cells (9.38% CD3+ CD4+ cells and 87.9% CD3+ CD8+ T cells), and some CD3+ cells co‐expressed CD56+ (23.57% CD3+ CD56+ T cells) which were used or cryopreserved.

### 2.3 Synthesis of anti‐CD3× anti‐B7H3 Bispecific Antibody (B7‐H3Bi‐Ab) and arming of ATC

One milligram per mL of Anti‐B7H3 mAb (R&D System, Minneapolis, MN, USA) was reacted with 2 mg/mL sulfo‐SMCC. Then, 1 mg/mL of Anti‐CD3 (OKT3, eBioscience) was reacted with 2 mg/mL Traut's reagent. Both reaction mixtures were incubated for 1 hour at room temperature and removed excess cross linker by PD‐10 column. The anti‐B7H3‐SMCC and anti‐CD3‐SH were mixed and hetero conjugated at 4°C overnight. The hetero conjugation was resolved by SDS‐PAGE and stained with Gelcode Blue. The coupling rate was calculated by grayscale analysis of Western blot using ImagJ software (National Institutes of Health, Maryland, Bethesda, USA). Cryopreserved ATCs were then unfreezed, and armed with B7‐H3Bi‐Ab at a concentration of 150 ng/10^6^ cells at room temperature for 30 minutes. The cells were washed to eliminate unbound antibodies. The combination of OKT3 (150 ng/10^6^ cells) and Anti‐B7‐H3‐mAb (150 ng/10^6^ cells) preincubated ATCs were used as unarmed control ATCs. In addition, w added unactivated PBMCs to the experiment and target bladder cancer cells were incubated either with B7‐H3Bi‐Ab‐PBMC or unarmed PBMC at an effector‐to‐target (E/T) ratio of 10:1.

### 2.4 Cytotoxicity assay

Target cells were seeded in triplicate in 96‐well u bottom microplates at 1 × 10^4^/well with the addition of Anti‐B7‐H3Bi‐armed, unarmed ATC or ATC cells at an effector‐to‐target (E/T) ratio of 10:1. Both were then interacted at 37°C for one night. Cytotoxicity was measured with two measures. One is luciferase quantitative assay.^28^, ^29^, ^30^, ^31^, ^32^ The other is collecting cell free supernatants, and the cytotoxicity was mostly measured with Lactate dehydrogenase activity kit (Sigma‐Aldrich, St. Louis, MO, USA).

### 2.5 ELISA assay

The IFN‐γ and TNF‐α productions were detected by the human cytokine ELISA kits (eBioscience) according to the manufacturer's instructions.

### 2.6 Flow cytometric analysis

The anti‐CD69‐PE, anti‐CD8‐FITC, and anti‐CD3‐FITC were provided by eBioscience, anti‐human B7H3‐PE mAb, and mouse IgG1‐PE isotype antibody were provided by R&D System. The cells were analyzed by the flow cytometer (CytoFLEX, Beckman Coulter), and data were processed using the accompanying software (CytExpert, Beckman Coulter, BeiJing, China).

### 2.7 Cytotoxicity of ATC from bladder cancer patients

In addition, peripheral blood mononuclear cells (PBMCs) were separated immediately using the method described previously from bladder cancer patients. And T cells were cultured and armed B7‐H3Bi‐Ab in the same manner as described above. The killing effect was proved by cytotoxicity assay, ELISA assay, and Flow cytometric analysis as described above.

### 2.8 Effect of anti‐B7‐H3 mAb and B7‐H3Bi‐Ab on bladder cancer cell proliferation

For evaluating Anti‐B7H3‐mAb on the proliferation of bladder tumor cell, the bladder cancer cells were seeded in a 96‐well flat plate and then 100 μL of different concentration gradient of B7H3 antibody (0.1, 1, 10 μg/mL) were added to wells and incubated at 37°C for 71 hours. Then, removed the medium and added 100 μL fresh medium which contains 10 μL Cell Counting Kit‐8 to each well and incubated for an additional 1 hour. The absorbency of bladder cancer cells was measured at 450 nm by Microplate reader after incubation. The proliferation of bladder cancer cells was assessed by the absorbance values. The same method described above is used for evaluating B7‐H3Bi‐Ab on bladder tumor cell proliferation.

## 3 RESULTS

### 3.1 Verify of B7‐H3 expression on human bladder cancer cells

**Figure 1:**
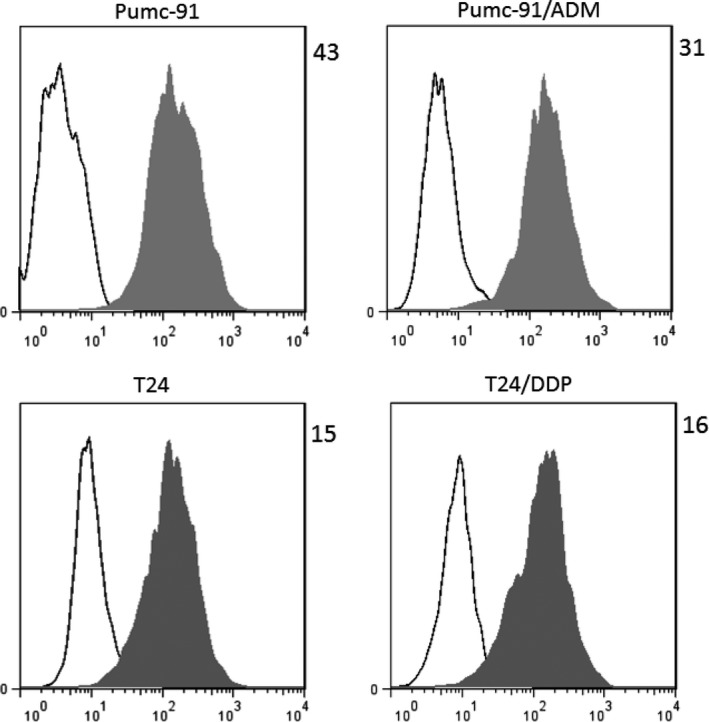
B7‐H3 expression on human bladder cancer cells. B7‐H3 expression was evaluated by flow cytometry on four bladder cancer cells (T24, T24/DDP, Pumc‐91, and Pumc‐91/ADM). Shaded histogram represents cells stained with anti‐B7‐H3 mAb and un‐shaded histogram represents cells stained with the control mouse IgG1. Mean Fluorescent Intensity (MFI) values obtained from anti‐B7‐H3 mAb staining divided by the control isotype staining are marked in the top right corner of the histogram

The expression of B7‐H3 on human bladder tumor cells was detected by FACS analysis. Mean Fluorescence Intensity (MFI) value was calculated by dividing anti‐human B7‐H3 mAb staining by control antibody staining, and marked in the top right corner of the histogram. As shown in Figure 1, B7‐H3 was highly expressed on four bladder cancer cells, including T24, and Pumc‐91 cells and their chemotherapeutic drug‐resistant counterparts, T24/DDP, and Pumc‐91/ADM.

### 3.2 Preparation and characterization of B7‐H3Bi‐Ab and ATCs

**Figure 2:**
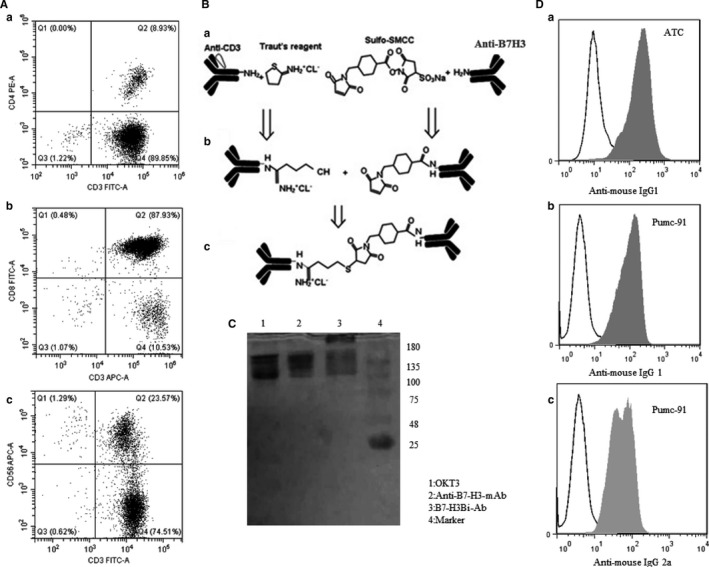
General program for B7‐H3Bi‐Ab and analyze of ATC. A, The molecule expression on ATC analyzed by flow cytometry. B, General program for the generation of B7‐H3Bi‐Ab. C, The concentrations of B7‐H3Bi‐Ab were measured by Coomassie blue staining of SDS‐gel. D, The assay of B7‐H3Bi‐Ab by flow cytometry

To produce a large quantity of T cells, PBMCs isolated from peripheral blood of healthy people were stimulated by ant‐CD3 mAb and IL‐2 for 13 days. CD3 expression was measured by FACS analysis. As shown in Figure 2A, ATC contained of CD3+ CD8+ T cells. In general, the above data indicate that cells mainly consist of ATCs and a small part of NK cells.

Simultaneously, the bispecific antibody B7‐H3Bi‐Ab was hetero‐conjugated by anti‐human B7‐H3 mAb and OKT3 (Figure 2B). The equimolar concentrations of binding product were measured by Coomassie blue (Figure 2C). The coupling rate is 24.5% calculated by ImageJ software. Then, we detected whether B7‐H3Bi‐Ab could recognize both CD3 on ATC and B7‐H3 on bladder cancer cells. ATC and pumc‐91 were stained with B7‐H3Bi‐Ab or a combination of OKT3 and anti‐human B7‐H3 mAb. Anti‐mouse IgG2a‐PE was detected the CD3 moiety of B7‐H3Bi‐Ab and anti‐mouse IgG1‐PE was detected the B7‐H3 moiety of B7‐H3Bi‐Ab (Figure 2D).

### 3.3 Cytotoxicity effects of B7‐H3Bi‐Ab‐armed ATC on bladder cancer cells

**Figure 3:**
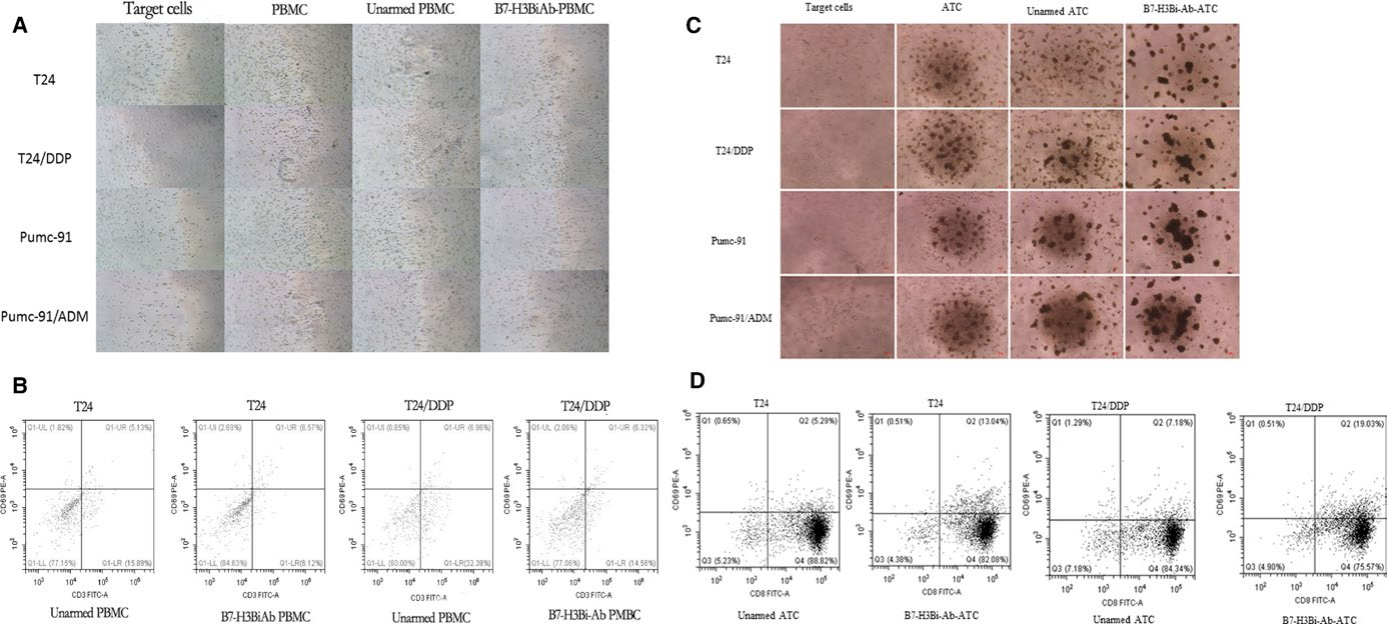
Cytotoxity effects of B7‐H3Bi‐Ab‐armed ATC on human bladder cancer cells. A, Target bladder cancer cells were incubated either with B7‐H3Bi‐Ab‐PBMC or unarmed PBMC at an E/T ratio of 10:1 for 18 h. Real‐time photographs of each bladder cancer group were taken at ×200 magnification. B, Expressions of CD69 on B7‐H3Bi‐Ab‐PBMC or unarmed PBMC were detected by flow cytometry after 18 h coculture with T24, T24/DDP. C, Target bladder cancer cells were incubated either with B7‐H3Bi‐Ab‐ATC or unarmed ATC at an E/T ratio of 10:1 for 18 h. Real‐time photographs of each bladder cancer group were taken at ×200 magnification. D, Expressions of CD69 on B7‐H3Bi‐Ab‐ATC or unarmed ATC were detected by flow cytometry after 18‐hour coculture with T24 and T24/DDP

According to the data provided by the literature,^21^, ^28^, ^29^, ^30^ 150 ng/10^6^ cells was chosen as the concentration of B7‐H3Bi‐Ab, ATC mixed with OKT3 and anti‐B7‐H3 mAb were used as unarmed ATC controls. Both were interacted at 37°C for 18 hours. Real‐time photographs of each bladder cancer group were taken at ×200 magnification (Figure 3A). Furthermore, FACS analysis showed increased CD69 expression on B7‐H3Bi‐Ab‐ATC over their unarmed ATC counterpart after 18‐hour incubation with T24 and T24/DDP cells (Figure 3B). CD69 has been known as an early activation marker of lymphocytes and has critical functions in immune responses.^33^ Increased CD69 expression on B7‐H3Bi‐Ab‐ATC proved that ATC was activated by B7‐H3Bi‐Ab. We used the same experiment in unactivated PBMCs. The result is not good because unactivated PBMC contains few T cells.

### 3.4 Cytokine production by B7‐H3Bi‐Ab‐ATC

**Figure 4:**
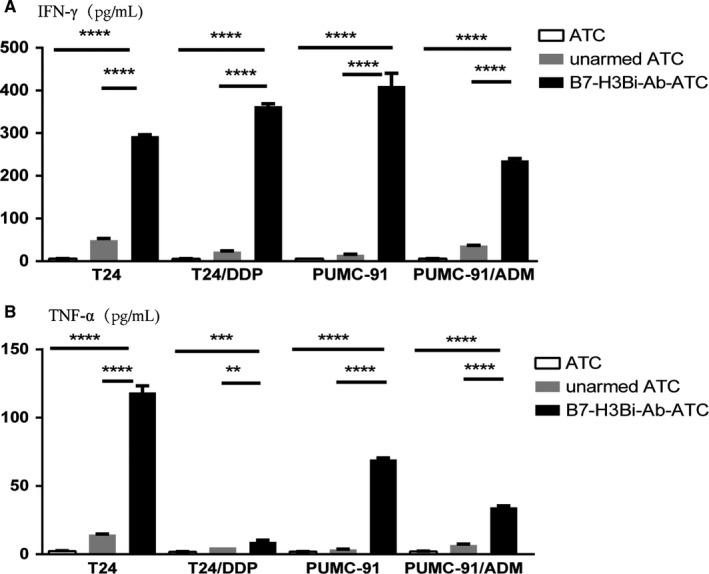
The secretion of IFN‐γ (A) and TNF‐α (B) by B7‐H3Bi‐Ab‐ATC against human bladder cancer cells. Supernatants of cocultures were collected as previously described and analyzed by ELISA kits. Image is a representative experiment of at least three experiments. *P < 0.05, **P < 0.01, ***P < 0.001, ****P < 0.0001

To analyze the level of cytokines, cell culture supernatants were collected for the IFN‐γ and TNF‐α productions. A significant increase was observed in IFN‐γ (Figure 4A) and TNF‐α (Figure 4B) secretion by B7‐H3Bi‐Ab‐ATC over their unarmed ATC counterpart when ATC were cultured with T24, T24/DDP, Pumc‐91, Pumc‐91/ADM. It is interesting that unarmed ATC also showed substantial IFN‐γ and TNF‐α production when cocultured with T24, T24/DDP, Pumc‐91, Pumc‐91/ADM.

### 3.5 Determination of the cytotoxicity of ATC

**Figure 5:**
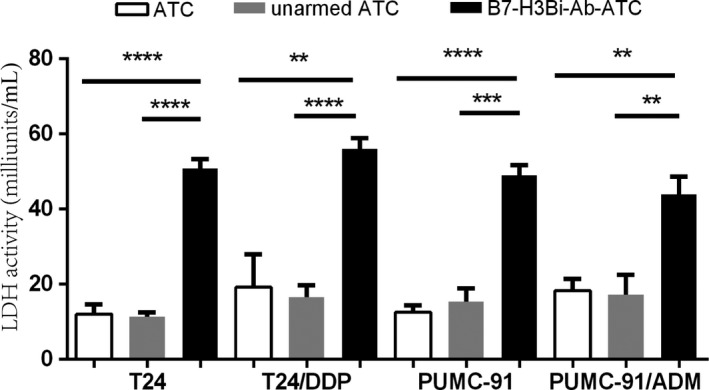
Lactate dehydrogenase activity by B7‐H3Bi‐Ab‐ATC against human bladder cancer cells. Supernatants were harvested and analyzed for cytokine levels by lactate dehydrogenase kit. Image is a representative experiment of at least three experiments. *P < 0.05, **P < 0.01, ***P < 0.001, ****P < 0.0001

Since lactate dehydrogenase (LDH) is a fairly stable enzyme, it has been widely used to evaluate the presence of damage and toxicity of cells. Here we collected the supernatants of cocultures. Consequently lactate dehydrogenase activity kit was used to determine the cytotoxicity. As shown in Figure 5, LDH activity was increased significantly in B7‐H3Bi‐Ab‐ATC compared with unarmed ATC counterparts when cocultured with four bladder cancer cell lines.

### 3.6 Luc cells for the determination of the cytotoxicity

**Figure 6:**
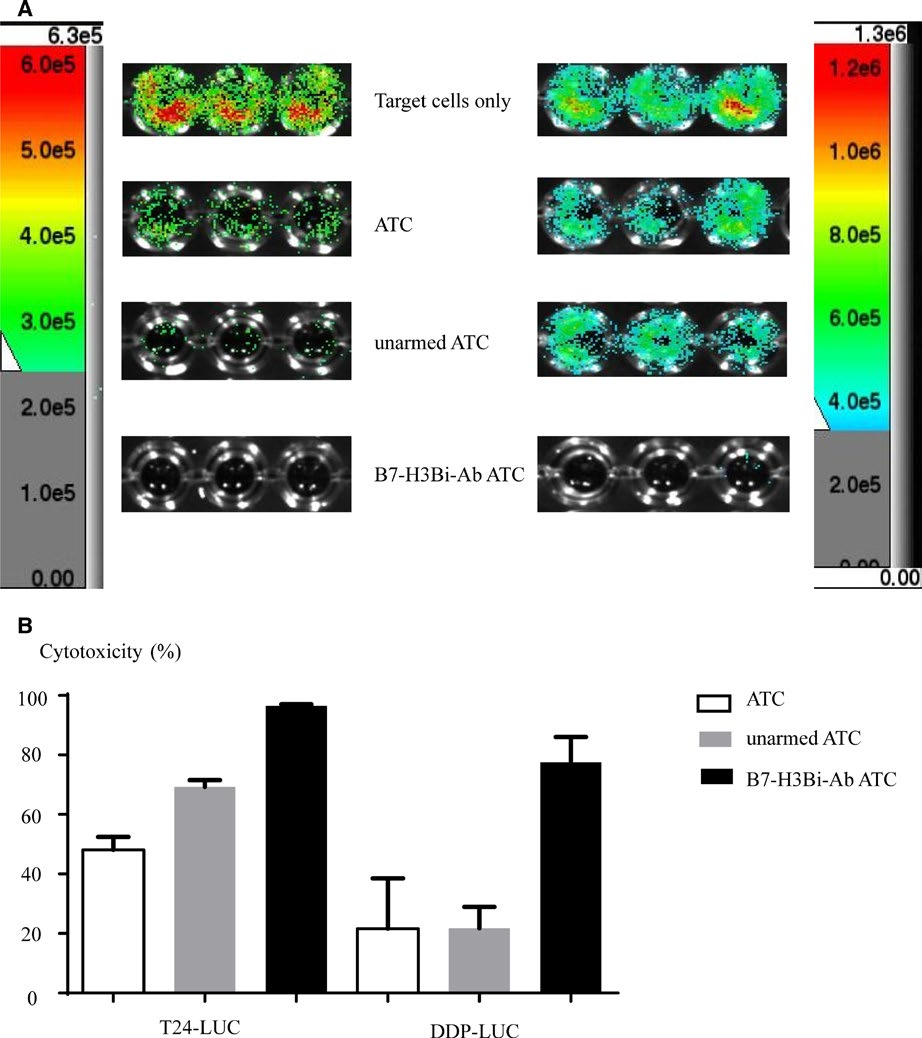
A, Bioluminescence images of T24‐luc cells and T24/DDP‐luc cells cultured with B7‐H3Bi‐Ab‐ATC or unarmed ATC cells. B, Bioluminescence image signal was measured by animal imaging system, and cytotoxicity assays were calculated at the E/T ratio of 10:1

To determine the killing rate accurately, stable bladder cancer cell line T24 and cisplatin resistant cell line T24/DDP that expressed luciferase reporter were generated (unpublished data). To determine the cytotoxicity, T24‐Luc and T24/DDP‐Luc cells were cultured with ATC, unarmed‐ATC, and B7‐H3Bi‐Ab‐ATC, respectively. The bioluminescent images were reflected the number of living T24‐luc and T24/DDP‐luc cells (Figure 6A). Bioluminescent image signal was measured by animal imaging system, and the cytotoxicity was calculated (Figure 6B). The bioluminescent images clearly showed that fluorescence in B7‐H3Bi‐Ab‐ATC cell was less than that in their unarmed ATC counterparts.

### 3.7 Cytotoxicity of ATC from bladder cancer patients

**Figure 7:**
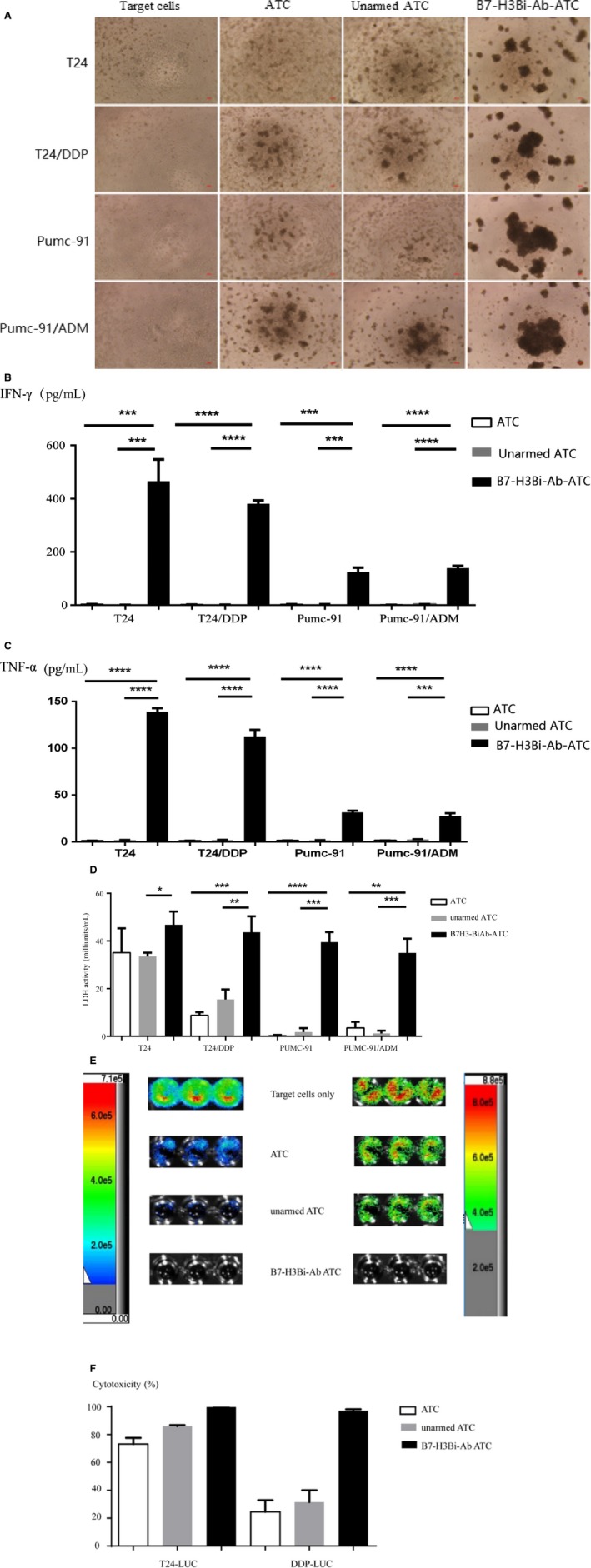
Cytotoxicity of ATC from bladder cancer patients. Target bladder cancer cells were incubated either with B7‐H3Bi‐Ab‐ATC or unarmed ATC at the E/T ratio of 10:1 for 18 h. (A) Real‐time photographs of each bladder cancer group were taken at ×200 magnification. Supernatants were collected after one night and analyzed for IFN‐γ (B) and TNF‐α production (C) and LDH activity (D). (E) Bioluminescence images of T24‐luc cells and DDP‐luc cells after incubation with B7‐H3Bi‐Ab‐ATC or unarmed ATC cells. (F) Bioluminescence image signal was measured by animal imaging system, and the cytotoxicity assays were calculated. *P < 0.05, **P < 0.01, ***P < 0.001,****P < 0.0001

To determine whether B7‐H3Bi‐Ab can effectively target the patients with bladder cancer, ATC isolated from the peripheral blood of bladder cancer patients were armed with B7‐H3Bi‐Ab and the above experiment was repeated. As Figure 7A shows, real‐time photographs of each bladder cancer group were taken at ×200 magnification. Significant increases were detected in IFN‐γ (Figure 7B) and TNF‐α (Figure 7C) secretion and LDH activity (Figure 7D) in B7‐H3Bi‐Ab‐ATC compared with their unarmed ATC counterpart when ATC were cocultured with T24, T24/DDP, Pumc‐91, Pumc‐91/ADM, respectively (*P *< 0.05). Similar results were observed when T24‐Luc (Figure 7E) and T24/DDP‐Luc (Figure 7F) were used.

### 3.8 No effect of anti‐B7‐H3 mAb and B7‐H3Bi‐Ab on bladder cancer cell proliferation

**Figure 8:**
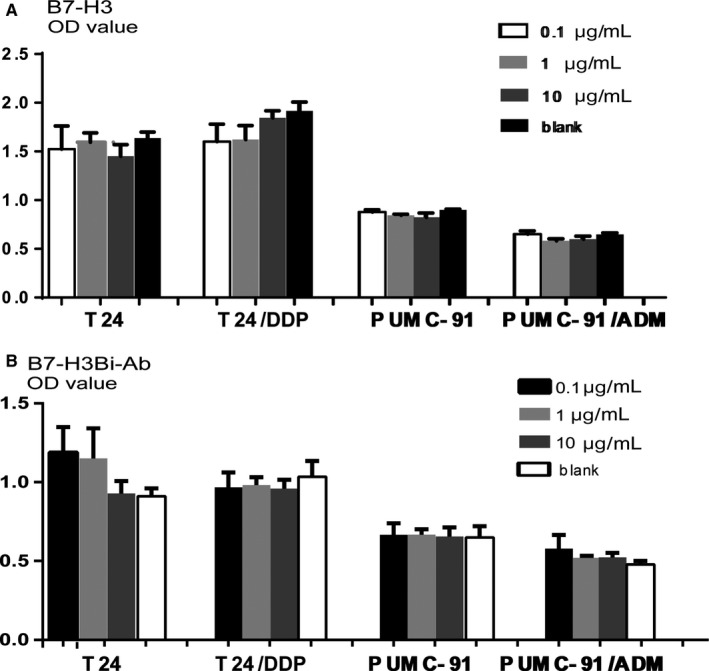
Effects of B7‐H3 (A) and B7‐H3Bi‐Ab (B) on the proliferation of bladder cancer cells. Bladder cancer cells were treated with or without B7‐H3 or B7‐H3Bi‐Ab for 72 h, and the proliferation of cells was assessed by CCK8 assay with the OD value was measured at 450 nm

In order to clarify whether anti‐B7‐H3 mAb or B7‐H3Bi‐Ab affect the proliferation of bladder cancer cells, the bladder cancer cells were cultured under different concentration of anti‐B7‐H3 mAb (Figure 8A) or B7‐H3Bi‐Ab (Figure 8B) for 71 hours. Then, the medium was removed and one hundred microliters of fresh medium containing Cell Counting Kit‐8 reagent was added to each well and incubated for an additional 1 hour. Even at the concentration of 10 μg/mL, anti‐B7‐H3 mAb or B7‐H3Bi‐Ab had no influence on the proliferation of bladder cancer cells.

## 4 DISCUSSION

In our country, bladder cancer is the most common malignancy of the urinary system. Chemotherapy is the main treatment of bladder cancer after surgery, but the occurrence of chemotherapy resistance often leads to chemotherapy failure, a serious threat to the lives of patients.

B7‐H3, a newly discovered member of the B7 family, is often overexpressed in a variety of types of human cancers, and its overexpression is closely correlated with survival, prognosis, or grade of tumor.^20^ Moreover, B7‐H3 has been demonstrated to mediate T‐cell proliferation and interferon‐γ production.^34^ Targeting B7‐H3 with monoclonal antibodies for the advanced‐stage central nervous system cancer in children has achieved effective results,^20^ making B7‐H3 a promising therapeutic target for cancer.

Our experiment used bispecific antibodies B7‐H3Bi‐Ab, redirecting T cells to bladder cancer cells. The results showed that ATC with B7‐H3Bi‐Ab had significant cytotoxic activity on human bladder cancer cells compared with ATC alone and unarmed ATC, probably due to the secured secreted higher level of IFN‐γ and TNF‐α. In addition, FACS analysis showed increased CD69 expression on B7‐H3Bi‐Ab‐ATC over their unarmed ATC counterpart. CD69 has been known as an early activation marker of T cells. CD69 expression is rapidly induced on the surface of T lymphocytes after TCR/CD3 engagement.^35^ Therefore, our results indicate that arming with B7‐H3Bi‐Ab triggers ATC activation and tumor cell killing.

To efficiently analyze the activity of ATC armed with B7‐H3Bi‐Ab to target tumor cells, T24‐luc cell and T24/DDP‐Luc cell lines were used to repeat the coculture experiments described above. All results showed that B7‐H3Bi‐Ab‐ATC could kill B7‐H3‐positive bladder cancer cells via the CD3‐B7‐H3 bridge. B7‐H3Bi‐Ab‐ATC still showed killing effect on T24/DDP‐Luc, indicating B7‐H3Bi‐Ab would be benefit in the field of targeting drug‐resistance tumor cells.

Our study showed that ATC from bladder patients combined with the B7‐H3Bi‐Ab had a strong killing effect on bladder cancer cells. This finding suggests a more convenient condition for the treatment of bladder cancer patients.

In summary, this study shows that B7‐H3Bi‐Ab enhances the ability of ATC to kill bladder cancer cells, suggesting that the development of bispecific antibodies favors cancer cell targeted therapy. Our study will provide a novel strategy for the immunotherapy of bladder cancer.

## CONFLICT OF INTEREST

None declared.
